# ABT-751 Induces Multiple Anticancer Effects in Urinary Bladder Urothelial Carcinoma-Derived Cells: Highlighting the Induction of Cytostasis through the Inhibition of *SKP2* at Both Transcriptional and Post-Translational Levels

**DOI:** 10.3390/ijms22020945

**Published:** 2021-01-19

**Authors:** Seyedeh Zahra Dehghanian, Cheng-Tang Pan, Jasmine Marianne Lee, Yow-Ling Shiue

**Affiliations:** 1 Institute of Biomedical Sciences, National Sun Yat-sen University, Kaohsiung, 70 Lienhai Rd, Kaohsiung 80424, Taiwan; s.z.dehghanian@g-mail.nsysu.edu.tw; 2 Institute of Precision Medicine, National Sun Yat-sen University, Kaohsiung 80424, Taiwan; pan@mem.nsysu.edu.tw; 3 Department of Mechanical and Electro-Mechanical Engineering, National Sun Yat-sen University, Kaohsiung 80424, Taiwan; 4 Johns Hopkins University, Baltimore, MD 21218, USA; jlee748@jhu.edu

**Keywords:** ABT-751, cytostasis, *S-phase kinase associated protein 2*, AKT, cyclin-dependent kinase inhibitors

## Abstract

The objective was to investigate the anti-cancer effects and underlying molecular mechanisms of cytostasis which were activated by an anti-microtubule drug, ABT-751, in two urinary bladder urothelial carcinoma (UBUC)-derived cell lines, BFTC905 and J82, with distinct genetic backgrounds. A series of in vitro assays demonstrated that ABT-751 induced G_2_/M cell cycle arrest, decreased cell number in the S phase of the cell cycle and suppressed colony formation/independent cell growth, accompanied with alterations of the protein levels of several cell cycle regulators. In addition, ABT-751 treatment significantly hurdled cell migration and invasion along with the regulation of epithelial–mesenchymal transition-related proteins. ABT-751 triggered autophagy and apoptosis, downregulated the mechanistic target of rapamycin kinase (MTOR) and upregulated several pro-apoptotic proteins that are involved in extrinsic and intrinsic apoptotic pathways. Inhibition of autophagosome and autolysosome enhanced apoptosis was also observed. Through the inhibition of the NFκB signaling pathway, ABT-751 suppressed *S-phase kinase associated protein* 2 (*SKP2*) transcription and subsequent translation by downregulation of active/phospho-AKT serine/threonine kinase 1 (AKT1), component of inhibitor of nuclear factor kappa B kinase complex (CHUK), NFKB inhibitor alpha (NFKBIA), nuclear RELA proto-oncogene, NFκB subunit (RELA) and maintained a strong interaction between NFKBIA and RELA to prevent RELA nuclear translocation for *SKP2* transcription. ABT-751 downregulated stable/phospho-SKP2 including pSKP2(S64) and pSKP2(S72), which targeted cyclin-dependent kinase inhibitors for degradation through the inactivation of AKT. Our results suggested that ABT-751 may act as an anti-cancer drug by inhibiting cell migration, invasion yet inducing cell cycle arrest, autophagy and apoptosis in distinct UBUC-derived cells. Particularly, the upstream molecular mechanism of its anticancer effects was identified as ABT-751-induced cytostasis through the inhibition of *SKP2* at both transcriptional and post-translational levels to stabilize cyclin dependent kinase inhibitor 1A (CDKN1A) and CDKN1B proteins.

## 1. Introduction

Urinary bladder urothelial carcinoma (UBUC) is a common malignant disease, particularly in developed countries [1]. Genetic and environmental factors impact UBUC development [2]. Histologic grade, stage, tumor size and multiplicity are critical clinicopathologic features associating with its progression. Although surgical techniques and multimodal remedies have been intensively improved, 5-year survival rates for patients with muscle-invasive UBUC continue to be disappointing. Roughly 50% of patients ultimately evolve systemic disease [3]. Genetic heterogeneity identified in UBUC patients additionally confuses the application of general therapies [4]. Cell-cycle dysregulation causing unrestrained cell proliferation has been correlated with UBUC development [5]. Cisplatin-based chemotherapy which suppresses cell proliferation provides a survival benefit in advanced disease. However, cisplatin resistance frequently emerges after treatment for a period of time.

The mammalian cell cycle is a firmly regulated procedure regulated by the fluctuating actions of cyclin-dependent kinases (CDKs), which are activated by cyclins but inhibited by cyclin-dependent kinase inhibitors (CKIs). Distinct mechanisms including transcriptional and translational levels, together with post-translational modifications regulate the oscillating activity of CDKs. Degradation of CKIs by the ubiquitin-proteasome system plays crucial roles to promote cell growth [6]. Ubiquitin-mediated proteasomal degradation is an irreversible event that ensures a strict unidirectionality of the cell cycle, mediating the exact spatial and temporal proteolysis of the main components of the cell cycle machinery. Prognostic significance of one CKI, cyclin-dependent kinase inhibitor 1B (CDKN1B, also known as p27) expression in UBUC has been reported [7]. Accordingly, to search for novel modulation of cell cycle progression by targeting its components is a rational approach for UBUC treatment.

The implication of microtubules in mitosis and the clinical accomplishment of anti-microtubule medicines, make these dynamic organelles one of the most appealing targets for anticancer treatment [8]. Both docetaxel [9] and paclitaxel [10] treatments in UBUC were encouraging despite being associated with the notorious side effect of neurotoxicity and are frequently accompanied with elevated drug resistance and cross resistance with other chemotherapeutic agents [9,10,11]. A second-generation microtubule-binding agent, ABT-751, was developed to prevent these disadvantages and improve the clinical application [12], since ABT-751 is not a multi-drug resistance transporter substrate [13]. Phase I/II study indicated that ABT-751 has therapeutic potential in squamous non-small-cell lung cancer (NSCLC) [14]. Through binding to the colchicine binding site on β-tubulin and inhibiting polymerization of microtubules, ABT-751 also revealed anti-vascular activity in a rat model with subcutaneous tumors [15]. As a single treatment or in combination with standard cytotoxic or radiation, ABT-751 is active in several human tumor xenograft models [16,17]. Our previous studies discovered that ABT-751 induced microtubule dysregulation, DNA damage, cell cycle arrest, autophagy and apoptosis in hepatocellular carcinoma-derived cells [18,19]. DNA damage causes cell cycle arrest for DNA repair. Once the damage is repaired, the cell cycle continues. Yet excessive DNA damage may result in apoptosis or enhance carcinogenesis [20]. Therefore, we aimed to study the anticancer activities of ABT-751, emphasizing the molecular mechanisms of ABT-751-induced cytostasis in two distinct UBUC-derived cell lines, BFTC905 and J82.

## 2. Results

### 2.1. ABT-751 Decreased Cell Number in the S Phase of the Cell Cycle and Suppresses Colony Formation/Anchorage-Independent Cell Growth along with Alteration of the Expression Levels of Several Cell Cycle Regulators in BFTC905 and J82 Cells

**Figure 1:**
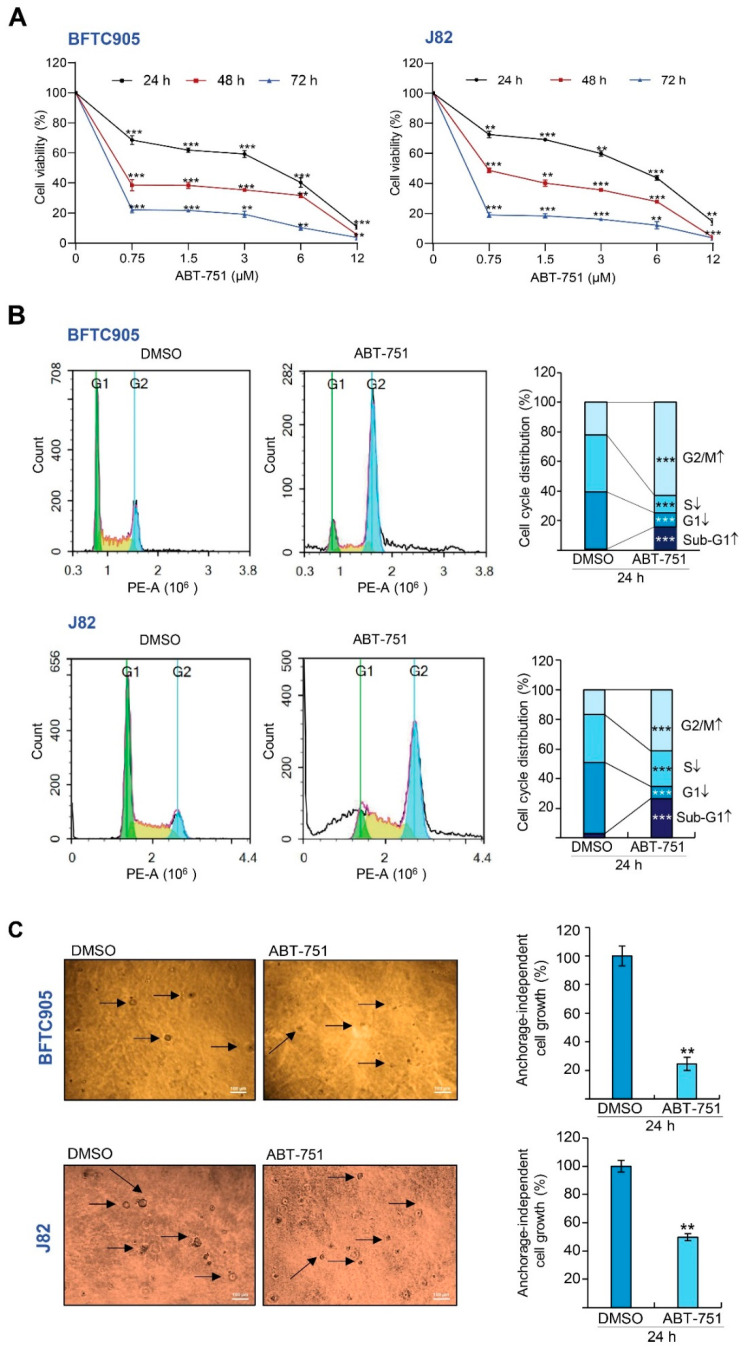

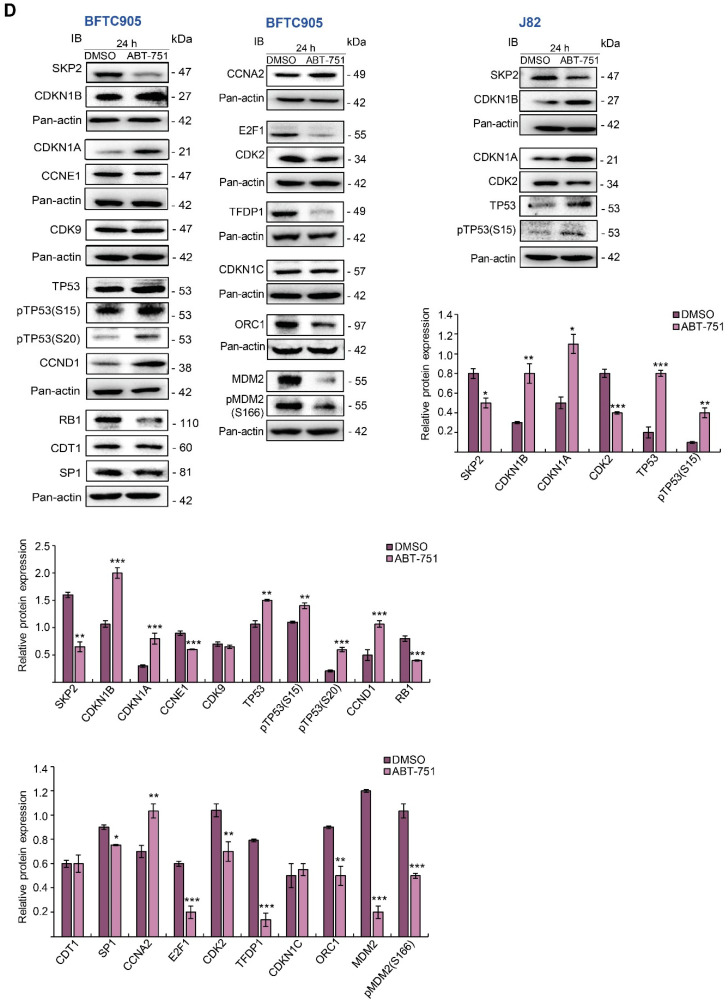
ABT-751 induces cytostasis and alters the protein expression levels of several cell cycle regulators in urinary bladder urothelial carcinoma (UBUC) cell lines. (**A**) UBUC-derived cell lines, BFTC905 and J82 were treated with different concentrations of ABT-751 or DMSO for the indicated time (24, 48 and 72 h), cell viability was next measured by the 3-(4, 5-dimethylthiazol-2-yl)-2, 5-diphenyltetrazolium bromide (MTT) assay. (**B**) The cells were treated with a specified concentration (BFTC905, 0.6 μM and J82, 0.7 μM) of ABT-751 for 24 h, and the cell cycle distribution was determined by flow cytometry. (**C**) After 7 days of ABT-751 treatment, colony formation and anchorage-independent cell growth were suppressed. Each arrow points to one colony. Scale bar: 100 µm. (**D**) The cells were treated with a specified concentration (BFTC905, 0.6 μM and J82, 0.7 μM) of ABT-751 for 24 h and the protein expression levels of several cell cycle regulators were determined by immunoblots. Relative protein expression of indicated proteins was quantified using ImageJ software (1.52a, NIH, USA). The results are expressed as the means ± SD. Pan-actin served as a loading control in each gel for immunoblot analysis. Statistical significance: * *p* < 0.05, ** *p* < 0.01, *** *p* < 0.001.

The 3-(4,5-Dimethylthiazol-2-yl)-2,5-diphenyltetrazolium bromide (MTT) assay identified that the IC_50_ of ABT-751 was >3 μM for 24 h, 0.6 μM for 48 h and 0.4 μM for 72 h in BFTC905 and >3 μM for 24 h, 0.7 μM for 48 h and 0.37 μM for 72 h in J82 cells (Figure 1A). The cell viabilities were 73.52% and 73.55% for BFTC905 and J82 cells, respectively, after treatments with ABT-751 for 24 h. In order to detect the effects of ABT-751 on BFTC905 and J82 cells as timely as possible, cells were harvested for analysis after treatments with 0.6 μM and 0.7 μM of ABT-751, respectively, for 24 h in most experiments except for time-course investigations. As shown in Figure 1B, after treatment with ABT-751 for 24 h, cell percentages in sub-G_1_ (*p* < 0.001) and G_2_/M (*p* < 0.001) phases were increased, however, cell percentages in G_1_ (*p* < 0.001) and S (*p* < 0.001) phases were decreased, suggesting that ABT-751 induced apoptosis, G_2_/M cell cycle arrest and suppressed DNA synthesis. Further treatment with ABT-751 for 7 days suppressed colony formation/anchorage-independent cell growth (Figure 1C), indicating that ABT-751 suppressed tumorigenesis in vitro. Among several examined cell cycle regulators, S-phase kinase associated protein 2 (SKP2), MDM2 proto-oncogene (MDM2), phospho-MDM2 at serine 166 (pMDM2(S166)), cyclin E1 (CCNE1), cyclin dependent kinase 2 (CDK2), RB transcriptional corepressor 1 (RB1), E2F transcription factor 1 (E2F1), transcription factor Dp-1 (TFDP1) and origin recognition complex subunit 1 (ORC1) were notably downregulated while tumor protein p53 (TP53), phospho-TP53 at serine 15 (pTP53(S15)), pTP53(S20), cyclin dependent kinase inhibitor 1A (CDKN1A), CDKN1B, CCND1 and CCNA2 protein levels were markedly upregulated in BFTC905 cells. On the other hand, SKP2 and CDK2 were downregulated while CDKN1B, CDKN1A, TP53, and pTP53(S15) protein levels were upregulated in both J82 and BFTC905 cell lines (Figure 1D). These observations suggested that ABT-751 may suppress cell proliferation together with the inhibition of SKP2 targeting CDKN1A and CDKN1B for proteasome-mediated degradation), E2F1/TFDP1 (*CCNE*1 transactivators) [21] and/or origin recognition complex subunit 1 (ORC1) [22] protein levels.

### 2.2. ABT-751 Inhibits Migration and Invasion in BFTC905 Cells

**Figure 2:**
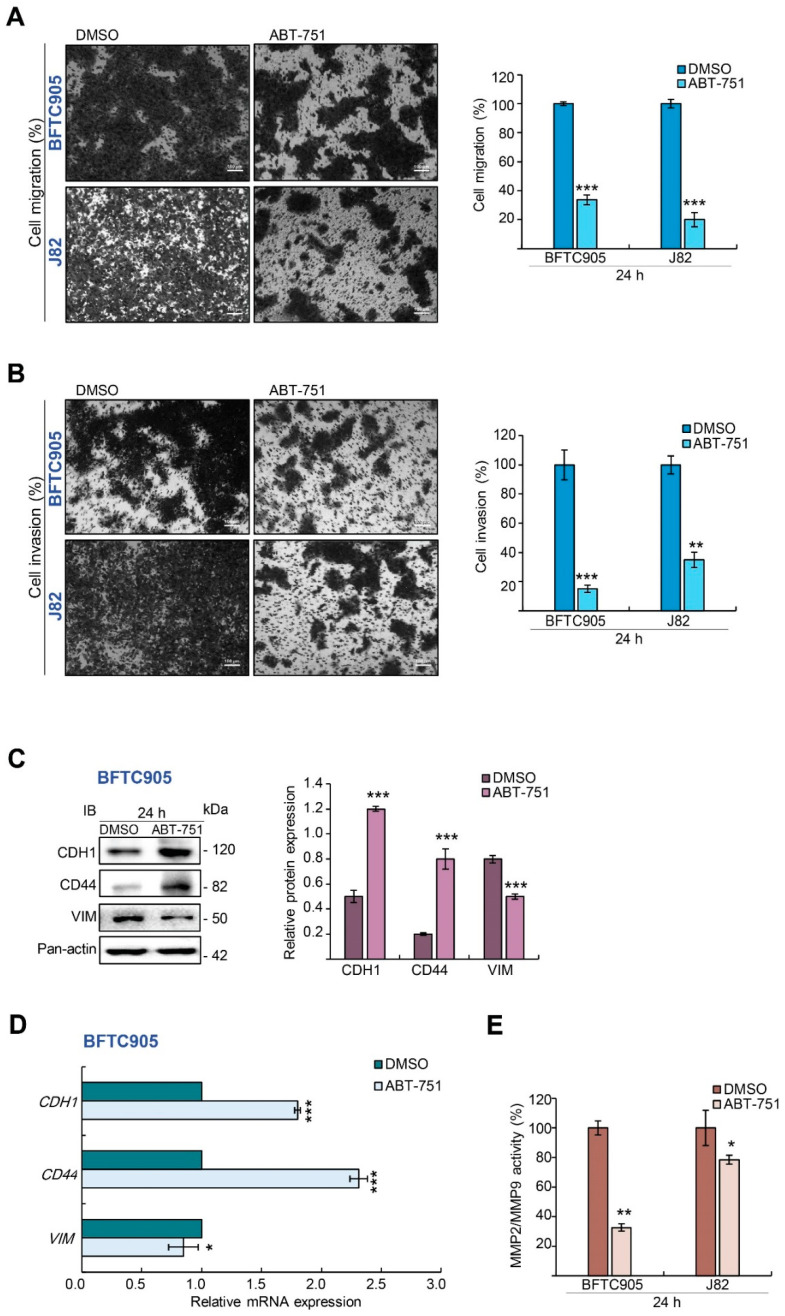
ABT-751 inhibits migration and invasion in UBUC cell lines. (**A**,**B**) The cells were treated with specified concentrations (BFTC905, 0.6 μM and J82, 0.7 μM) of ABT-751 for 24 h and cell migration and invasion were analyzed using the QCM ECMatrix Kit. Scale bar: 100 µm. (**C**,**D**) BFTC905 cells were treated with ABT-751 (0.6 μM) for 24 h; the levels of several migration and invasion-associated proteins and mRNA were examined by Immunoblots and quantitative RT-PCR, respectively. (**E**) Both UBUC-derived cell lines were treated with a specified concentration of ABT-751 (BFTC905, 0.6 μM and J82, 0.7 μM). Next, MMP2/MMP9 activity was analyzed by gelatinase activity assays. Relative protein expression of the indicated proteins was quantified using ImageJ software. The results are expressed in the means ± SD. Pan-actin served as a loading control in each gel for immunoblot analysis. Statistical significance: * *p* < 0.05, ** *p* < 0.01, *** *p* < 0.001.

Transwell migration and transwell invasion assays showed that treatment with ABT-751 for 24 h inhibited cell migration (*p* < 0.001; Figure 2A) and invasion (*p* < 0.001; Figure 2B) in BFTC905 and J82 cells. ABT-751 consistently upregulated cadherin 1 (*CDH*1) and *CD*44, whereas it downregulated *VIM* at mRNA and protein levels (Figure 2C,D) in BFTC905 cells. Moreover, ABT-751 inhibited MMP2/MMP9 activity in both cell lines (*p* < 0.01; Figure 2E). Therefore, in addition to impeding cell proliferation, ABT-751 further inhibits cell migration and invasion in vitro along with changing the expression levels of two epithelial–mesenchymal transition (EMT) markers, *CDH*1 and *VIM*, at the mRNA and protein levels and the activity of metastatic enzymes.

### 2.3. ABT-751 Induces Autophagy, Apoptosis and Inhibition of the Formation of Autophagosome Augments ABT-751-Induced Apoptosis in BFTC905 and J82 Cells

**Figure 3:**
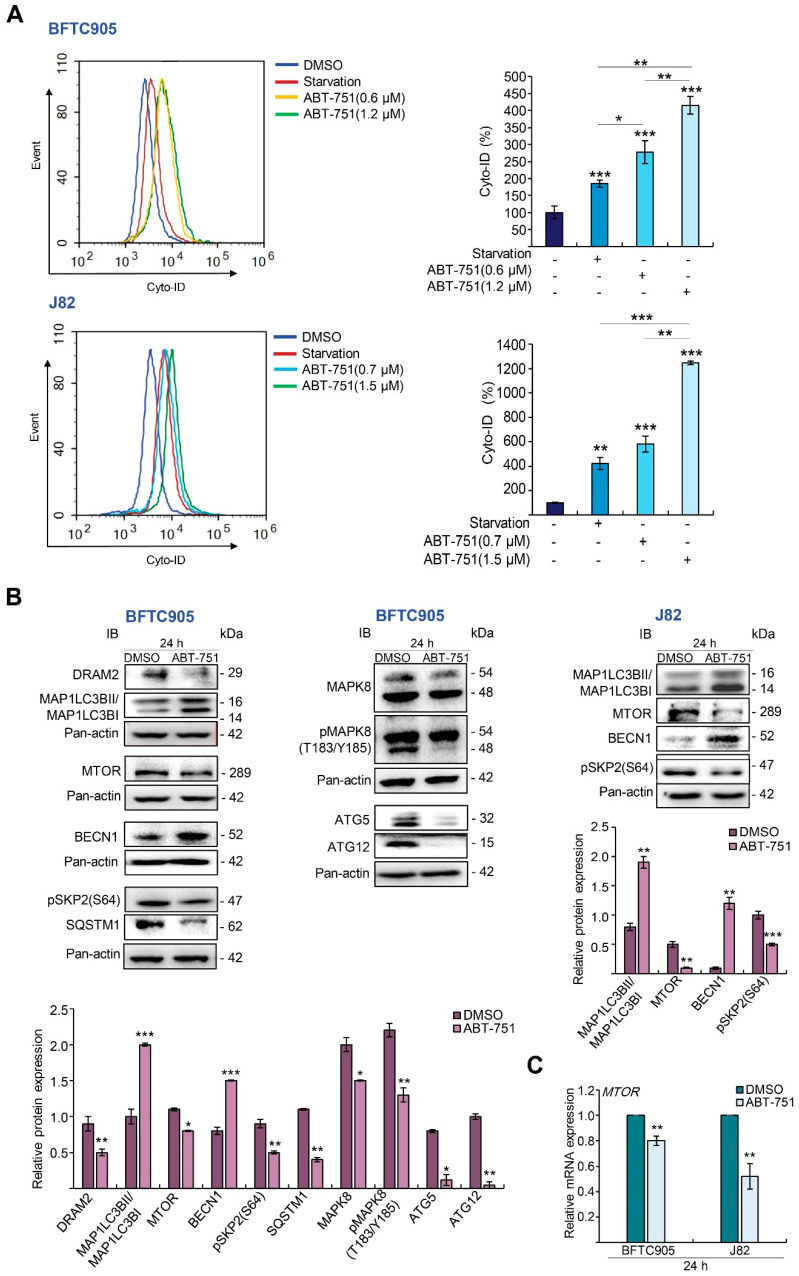

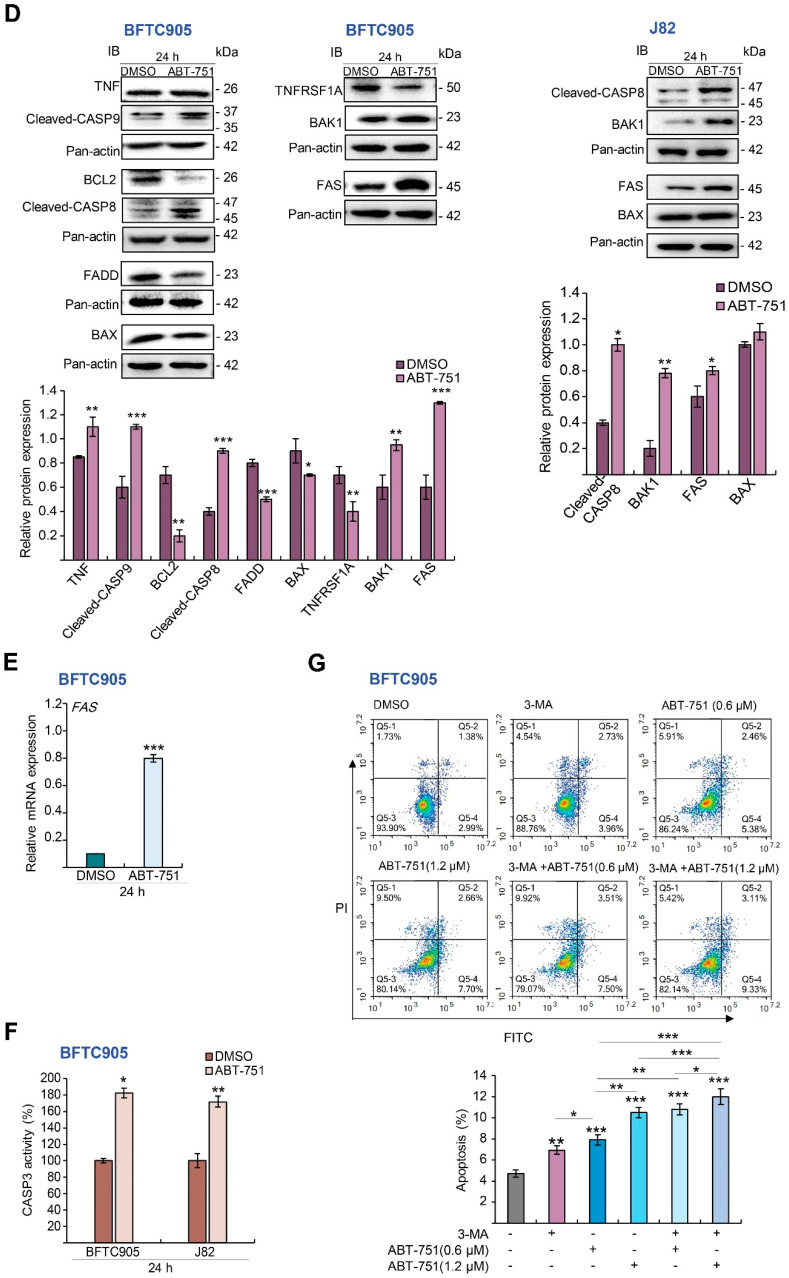

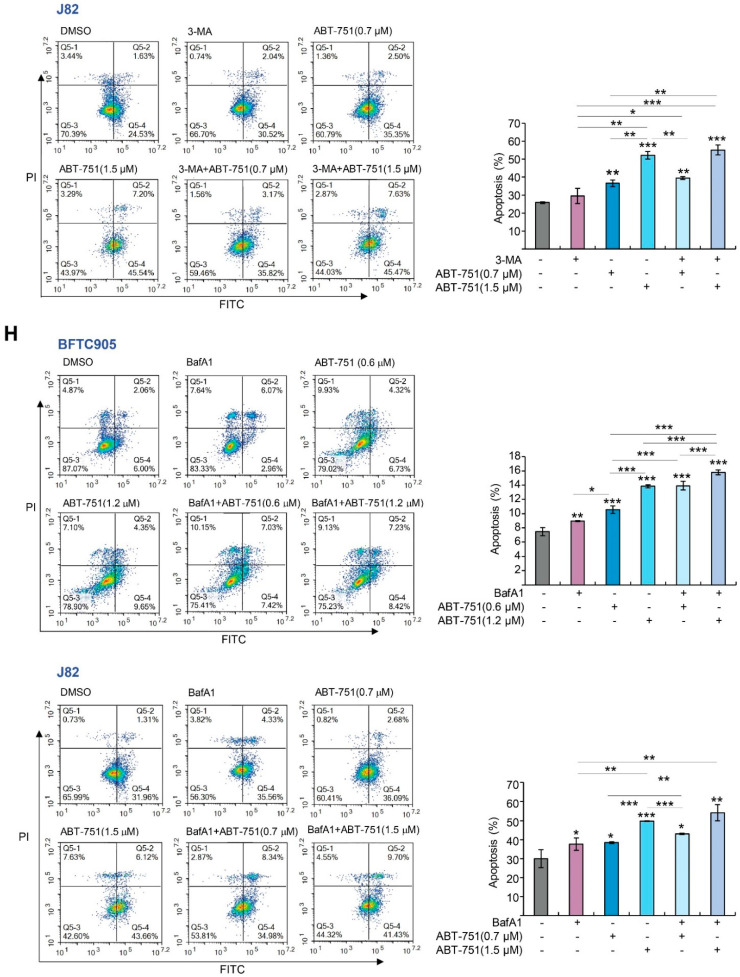
ABT-751 induces autophagy accompanied by the mechanistic target of rapamycin kinase (MTOR) downregulation and apoptosis via both extrinsic and intrinsic pathways in the UBUC cells. (**A**) BFTC905 cells (up) and J82 cells (down) were treated with different concentrations of ABT-751 (BFTC905, 0.6 and 1.2 μM; J82, 0.7 and 1.5 μM) for 24 h, and Autophagic flux was monitored by the Cyto-ID^®^ Autophagy Detection Kit; starvation (containing 2% fetal bovine serum (FBS) in culture medium) served as a positive control. (**B**) BFTC905 and J82 cells were treated with a specified concentration of ABT-751 (0.6 and 0.7 μM, respectively) and the levels of various autophagy-related proteins were investigated by immunoblots. (**C**) The mRNA level of *MTOR* was examined by quantitative RT-PCR in both BFTC905 and J82 cells. (**D**) The UBUC-derived cell lines were treated with the indicated concentration of ABT-751 (BFTC905, 0.6 μM and J82, 0.7 μM), and the levels of apoptosis-associated proteins were determined by immunoblots. (**E**) The mRNA level of *FAS* was determined by quantitative RT-PCR in BFTC905 cells, (**F**) CASP3 activity analysis was investigated by CASP3 colorimetric assay after ABT-751 treatment in both cell lines. (**G**) The cells were pretreated with an autophagosome inhibitor, 3-MA (3-methyladenine) (3 mM) for 8 h and treatment with a different concentration of ABT-751 (BFTC905, 0.6 and 1.2 μM; J82, 0.7 and 1.5 μM) for another 16 h, then the apoptotic cells were detected using Annexin V- fluorescein (FITC) staining and flow cytometric methods. (**H**) The percentage of Annexin V-positive cells was analyzed by Annexin V-FITC staining and flow cytometric methods in both UBUC cells that were pretreated with 20 nM of Bafilomycin A1 (BafA1, an autolysosome inhibitor) for 8 h and treated with different concentrations of ABT-751 (BFTC905, 0.6 and 1.2 μM; J82, 0.7 and 1.5 μM) for another 16 h. Relative protein expression of indicated proteins was quantified using ImageJ software. The results are expressed as the means ± SD. Pan-actin served as a loading control in each gel for immunoblot analysis. Statistical significance: * *p* < 0.05, ** *p* < 0.01, *** *p* < 0.001.

Treatment with ABT-751 induced autophagy compared to the control (*p* < 0.001) and starvation group (*p* < 0.001; positive control) in BFTC905 cells (Supplementary Materials Figure S1). ABT-751 stimulated autophagy in a dose-dependent manner, yet autophagy was reduced when prolonging the treatments from 2 to 16 h with the same concentration (0.6 μM), suggesting that it was a time-dependent decrease. Nevertheless, autophagy was increased at 2, 4 and 16 h after treatments compared to the control (Figure 3A; *p* < 0.001, Supplementary Materials Figure S2). ABT-751 also upregulated microtubule associated protein 1 light chain 3 beta II (MAP1LC3B-II)/I ratio and key autophagy mediator, beclin 1 (BECN1), in BFTC905 and J82 cells, while it downregulated DNA damage regulated autophagy modulator 2 (DRAM2), mechanistic target of rapamycin kinase (MTOR), pSKP2(S64), mitogen-activated protein kinase (MAPK8), pMAPK8(T183/Y185), sequestosome 1 (SQSTM1), autophagy related 5 (ATG5) and ATG12 protein (Figure 3B) and *MTOR* mRNA (Figure 3C) levels, indicating that ABT-751-induced autophagy accompanied by the induction of MAP1LC3B-II and BECN1 and the suppression of *MTOR* transcription and subsequent translation in two UBUC-derived cell lines. Treatment with ABT-751 for 24 h in BFTC905 cells upregulated tumor necrosis factor (TNF), Fas cell surface death receptor (FAS), BCL2 antagonist/killer 1 (BAK1), cleaved-caspase 8 (CASP8) and -CASP9 (Figure 3D) protein, *FAS* mRNA (Figure 3E) levels and CASP3 activity (Figure 3F), which signified that ABT-751 triggered both extrinsic and intrinsic apoptotic pathways. We also validated these aspects in another UBUC-derived cell line, J82, with distinct genetic backgrounds (Figure 3D–F). Dose–response experiments demonstrated that 3 mM of 3-methyladenine (3-MA, an autophagosome inhibitor) and 20 nM of Bafilomycin A1 (BafA1, an autolysosome inhibitor) treatments for 24 h were efficient to inhibit endogenous autophagy (Supplementary Materials Figures S3 and S4, respectively). These conditions were applied for further trials. Additional treatment with 3-MA and BafA1 augmented ABT-751-induced apoptosis (Figure 3G,H).

### 2.4. ABT-751 Suppresses SKP2 Transcription and Subsequent Translation through the Inhibition of AKT Serine/Threonine Kinase–Nuclear Factor Kappa B Signaling Pathway in BFTC905 and J82 Cells

**Figure 4:**
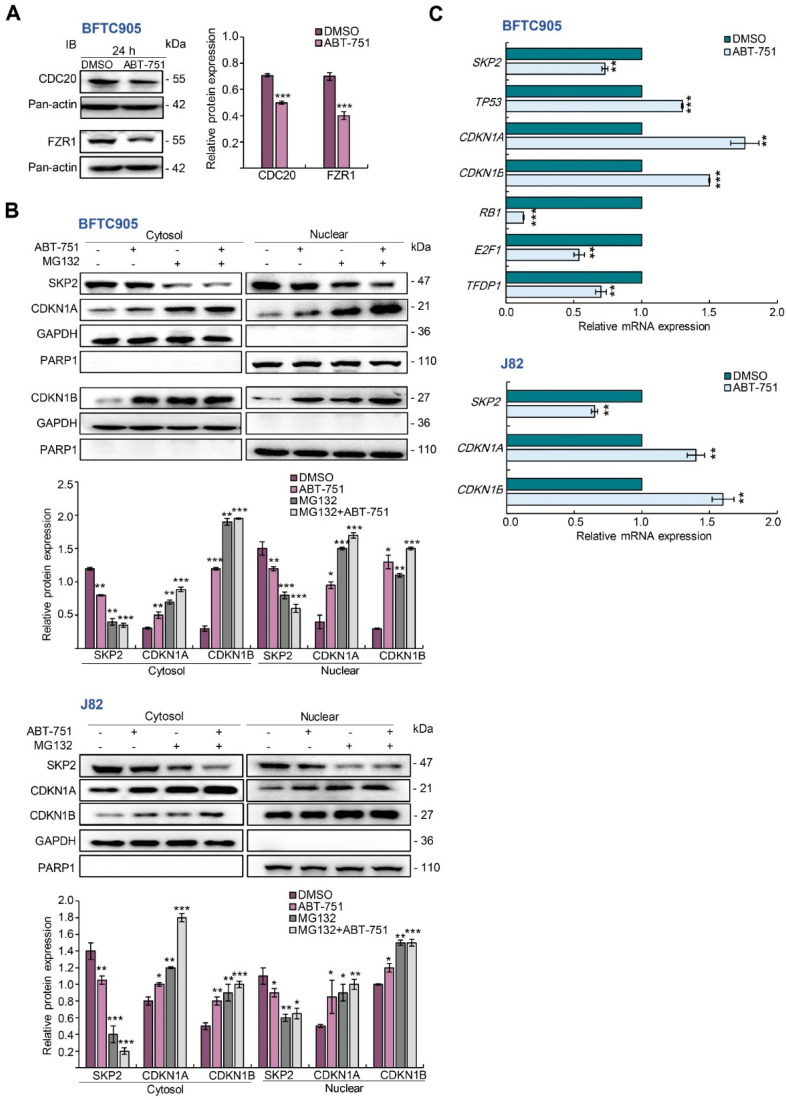

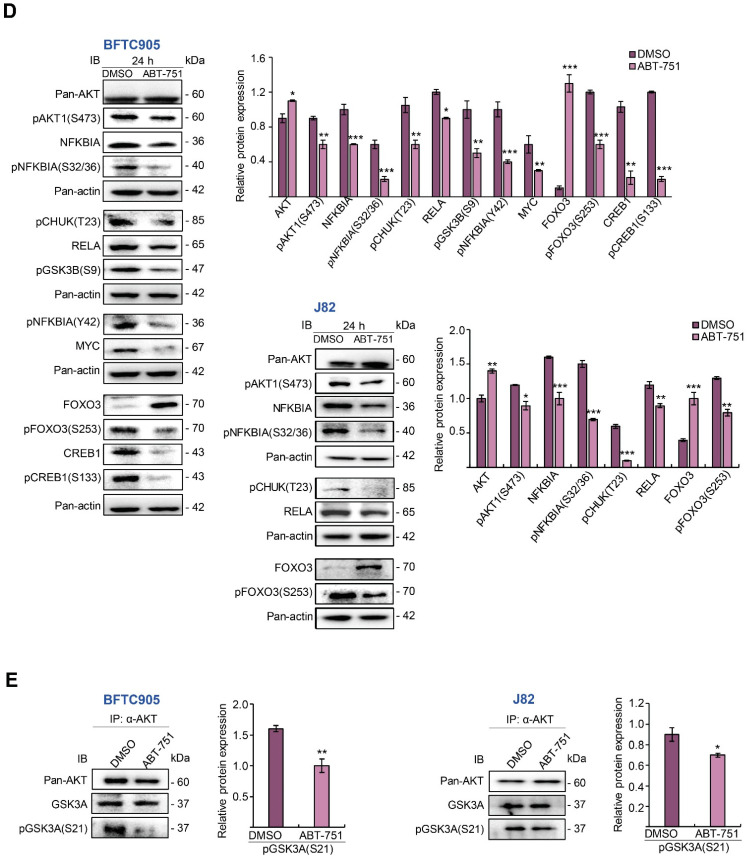

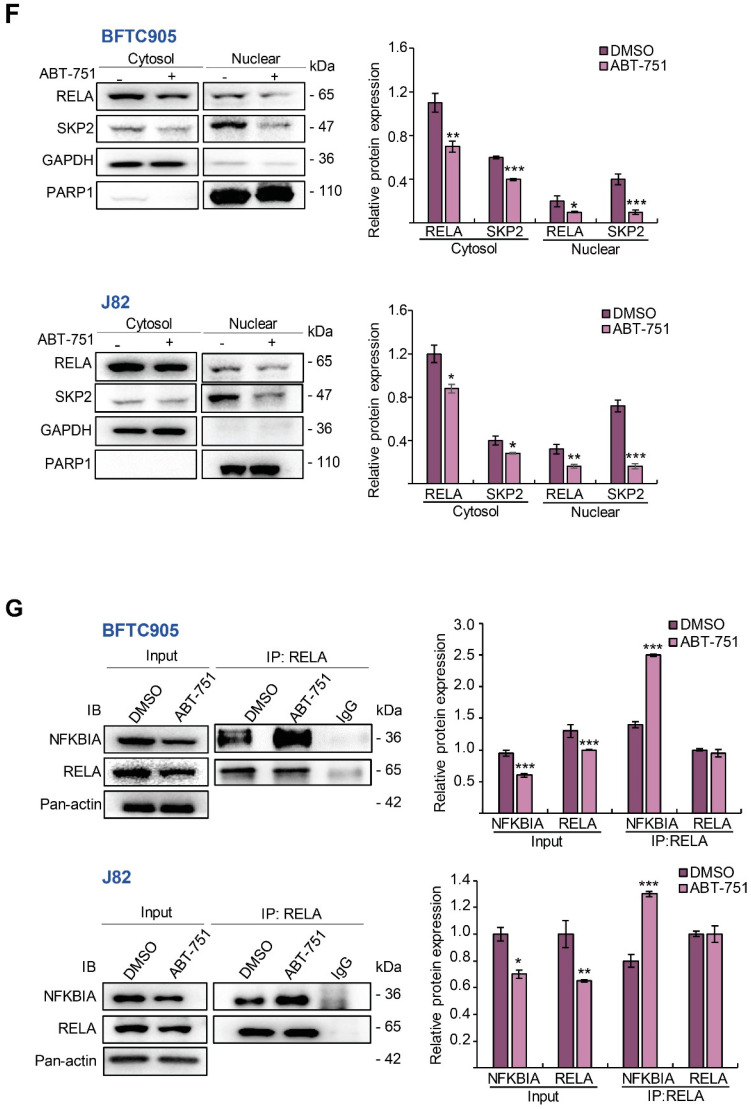

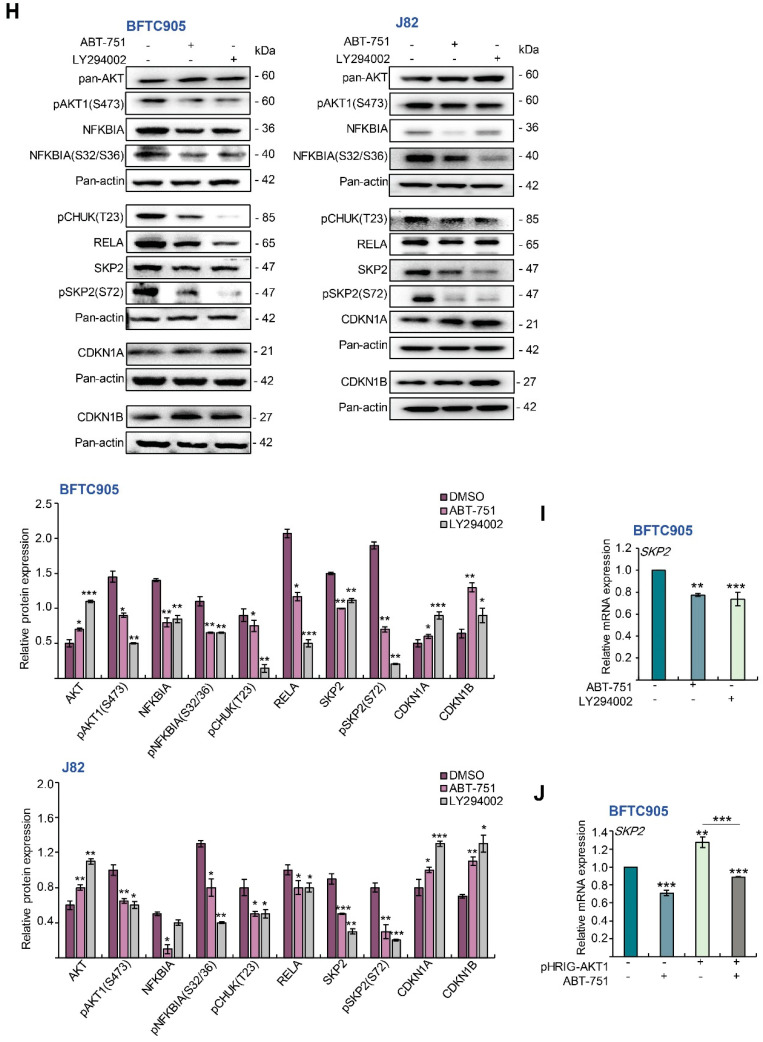
ABT-751 inhibits *SKP2* transcription and subsequent translation in UBUC cells through dysregulation of the AKT–CHUK–NFKBIA–NFKB signaling pathway. (**A**) BFTC905 cells were treated with ABT-751 (0.6 μM) for 24 h and the levels of the components of SKP2 E3 ligase (CDC20 and FZR1 proteins) were determined by immunoblots. (**B**) The cells were pretreated with 4 μM of MG132, a proteasome inhibitor, for 6 h, then treated with a specified concentration of ABT-751 (BFTC905, 0.6 μM and J82, 0.7 μM) for another 18 h. Next, nuclear/cytosol fractionation along with immunoblot analysis were performed. (**C**) The mRNA levels of *SKP2*, *TP*53, *CDKN*1*A*, *CDKN*1*B*, *RB*1, *E*2*F*1 and *TFDP*1 were analyzed by quantitative RT-PCR. (**D**) The expression levels of the *SKP2* gene regulator-associated proteins were determined by immunoblots in BFTC905 and J82 cells. Cells were treated with a specified concentration of ABT-751 (BFTC905, 0.6 μM and J82, 0.7 μM) for 24 h, (**E**) AKT1 kinase activity via a co-immunoprecipitation assay and (**F**) nuclear/cytosol fractionation along with immunoblot analysis were performed. (**G**) The cells were treated with a specified concentration of ABT-751 (BFTC905, 0.6 μM and J82, 0.7 μM) for 24 h, cell lysates were subjected to the co-immunoprecipitation assay with anti-RELA antibody. (**H**) Immunoblot analysis was used to compare the alterations of *SKP2*-regulatory protein levels after treatment with a PI3K-AKT inhibitor, LY294002 (10 μM) for 2 h and ABT-751 (BFTC905, 0.6 μM and J82, 0.7 μM) for 24 h. (**I**) BFTC905 cells were treated with ABT-751 (0.6 μM) or LY294002 (10 μM) and *SKP2* mRNA levels were analyzed by quantitative RT-PCR. (**J**) Transfection of a constitutive active AKT1 plasmid, pHRIG-AKT1, into BFTC905 cells for 24 h, suppressed AKT1-induced *SKP2* mRNA levels. Relative protein levels were quantified using Image J software. The results are expressed as the means ± SD. Pan-actin, GAPDH and PARP1 served as loading, cytosolic and nuclear controls, respectively, in each gel. Statistical significance: * *p* < 0.05, ** *p* < 0.01, *** *p* < 0.001.

Disruption of cytostasis is an early event of tumorigenesis, as we identified that ABT-751 downregulated *SKP2* mRNA and its corresponding protein levels in both UBUC-derived cell lines (Figure 1D). We next evaluated whether ABT-751-downregulated SKP2 protein levels were ubiquitin-dependent. However, ABT-751 treatments for 24 h were not able to increase two components of anaphase promoting complex (APC/C, a multi-subunit RING finger type E3 ligase targeting SKP2 for degradation), CDC20 and FZR1 protein levels in BFTC905 cells (Figure 4A), suggesting that ABT-751-suppressed SKP2 protein level was not produced through increase in the protein stability of CDC20 or FZR1. A dose–response experiment showed that treatments with 2, 4, 8 and 16 μM of MG132 (a proteasome inhibitor) for 6 h upregulated both CDKN1A and CDKN1B compared to the control. However, 4 μM of MG132 treatment is more effective to upregulate endogenous CDKN1B protein level (Supplementary Materials Figure S5). Thereafter, treatment with MG132 notably downregulated nuclear/endogenous and ABT-751-suppressed SKP2, whereas upregulated endogenous/nuclear and ABT-751-induced nuclear CDKN1A and CDKN1B protein levels in BFTC905 and J82 cells (Figure 4B). On the other hand, ABT-751 treatment for 24 h downregulated *SKP2* (*p* < 0.01), *RB*1 (*p* < 0.001), *E*2*F*1 (*p* < 0.01) and *TFDP*1 (*p* < 0.01) while it upregulated *TP*53 (*p* < 0.001), *CDKN*1*B* (*p* < 0.001) and *CDKN*1*A* (*p* < 0.01) mRNA levels in BFTC905 and/or J82 cells (Figure 4C), suggesting that ABT-751 may regulate *SKP2*, *CDKN*1*A*, *RB*1 and *E*2*F*1 transcription and following translation.

Since the NF-κB (NFKB) complex is one well-recognized transcription factor of the *SKP2* gene [23], related regulators were next examined. We found that ABT-751 also downregulated pAKT1(S473), pCHUK(T23), NFKBIA, pNFKBIA(S32/S36), RELA, pNFKBIA(Y42), GSK3B(S9), MYC, pFOXO3(S253), CREB1 and pCREB1(S133) protein levels (Figure 4D), signifying that ABT-751-inhibited *SKP2* transcription may occur through suppression of the phosphoinositide 3-kinase (PI3K)–AKT–CHUK–NFKB axis. The treatment of ABT-751 further decreased the AKT kinase activity as evidence by marked decrease in the interaction between AKT and pGSK3A(S21) proteins in BFTC905 and J82 cells (Figure 4E). Nuclear/cytosol fractionation and co-immunoprecipitation assays identified that nuclear RELA and SKP2 were notably downregulated (Figure 4F) and the interaction between NFKBIA and RELA remained strong compared to the recovered RELA after immunoprecipitation (immunoprecipitation (IP): anti-RELA antibody, immunoblot (IB): anti-NFKBIA antibody (Figure 4G)) after treatment with ABT-751 for 24 h in both BFTC905 and J82 cell lines. These results reinforced a reduction in the transcriptional activity of RELA by binding to NFKBIA in *SKP2* transcription after ABT-751 treatment.

Furthermore, treatment with a PI3K/AKT inhibitor, LY294002 in BFTC905 and J82 cells notably downregulated pAKT1(S473), pCHUK(T23), NFKBIA, pNFKBIA(S32/S36), RELA, SKP2, pSKP2(S72) while it upregulated CDKN1A and CDKN1B protein levels, similar to those that were treated with ABT-751 (Figure 4H). Moreover, treatment with LY294002 downregulated *SKP2* mRNA levels compared to the control, which is comparable to the effect of ABT-751 in BFTC905 cells (Figure 4I). Accordingly, ABT-751 suppressed *SKP2* transcription and thereafter translation, similar to a pan-AKT inhibitor, LY294002. Further exogenous expression of the constitutive active *AKT*1 gene in BFTC905 cells was upregulated (*p* < 0.01), while treatment with ABT-751 for another 24 h suppressed AKT1-upregulated (*p* < 0.001) *SKP2* mRNA levels (Figure 4J). Therefore, ABT-751 suppresses *SKP2* transcription and translation through the inhibition of the AKT–CHUK–NFKBIA–NFKB signaling pathway in BFTC905 and J82 cells.

### 2.5. ABT-751 Suppresses SKP2 via the Inhibition of AKT Signaling Pathway at Post-Translational Level in UBUC-Derived Cells

**Figure 5:**
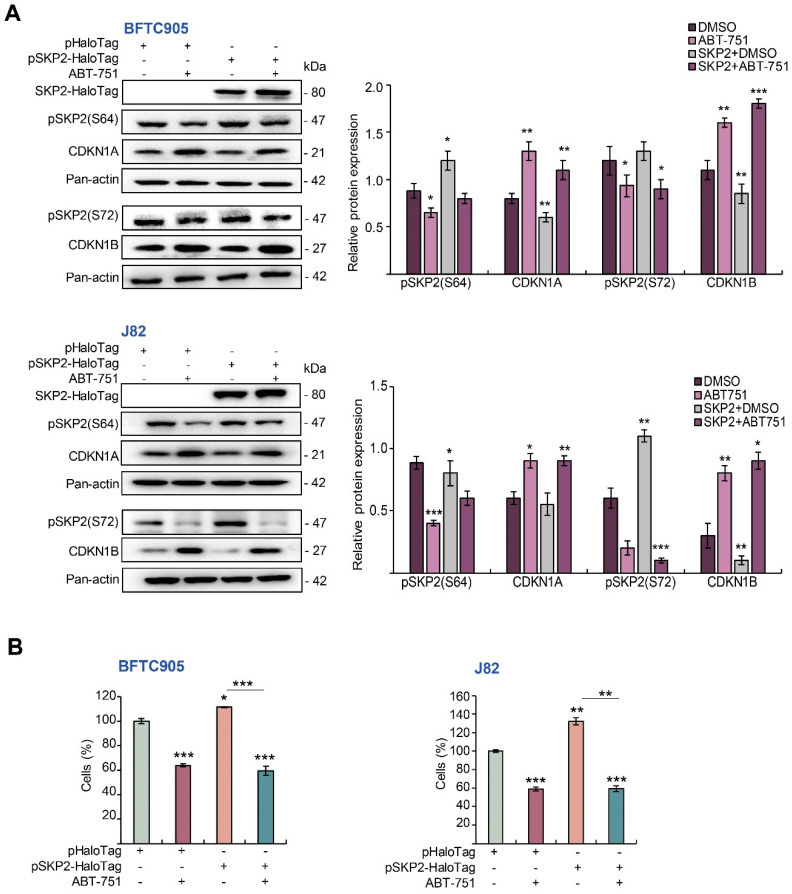

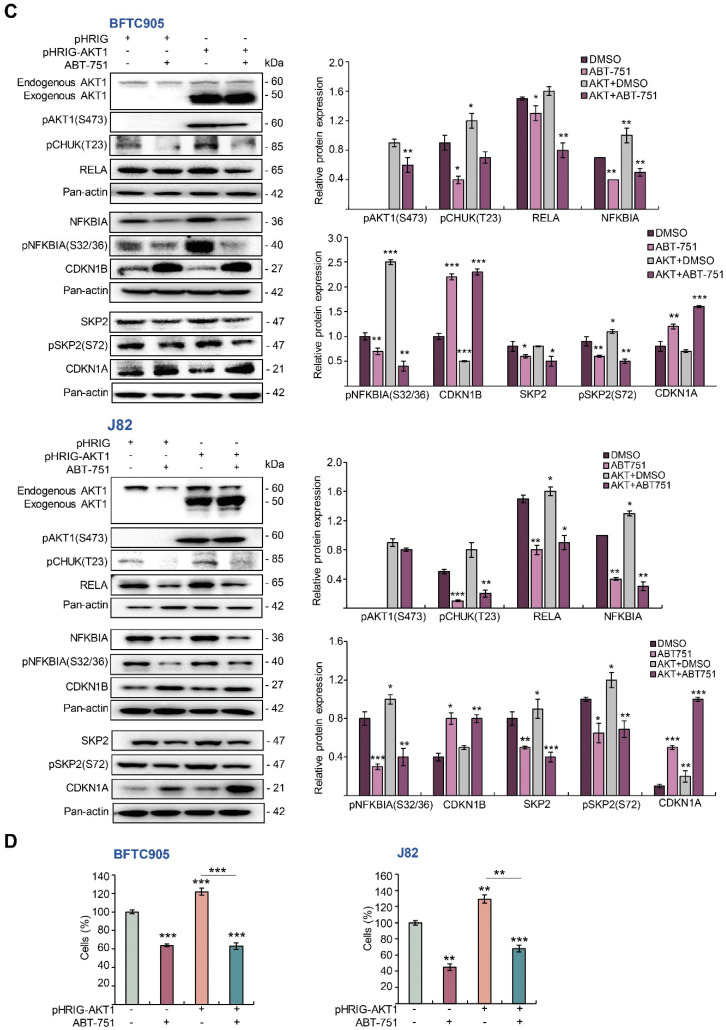
ABT-751 induces cytostasis by downregulation of stable/phospho-SKP2 proteins through the inhibition of the PI3K–AKT signaling pathway in UBUC-derived cell lines. BFTC905 and J82 cells were transfected with pHTC-HaloTag (as a control) or pSKP2-HaloTag. After 24 h, the transfected cells were treated with the indicated concentration of ABT-751 (BFTC905, 0.6 μM and J82, 0.7 μM). Next, (**A**) the expression levels of pSKP2(S72), pSKP2(S64), CDKN1A, and CDKN1B proteins were investigated by immunoblots and (**B**) a cell proliferation assay was conducted. BFTC905 and J82 cells were transfected with pHRIG (control) or pHRIG-AKT1. After 24 h, the transfected cells were treated with the indicated concentration of ABT-751 (BFTC905, 0.6 μM and J82, 0.7 μM), and (**C**) the expression levels of various AKT–CHUK–NFKBIA–NFKB signaling pathway-associated proteins were measured by immunoblots, and a cell proliferation assay was conducted (**D**). Relative protein levels were quantified using ImageJ software. The results are expressed as the means ± SD. Pan-actin served as the loading control in each gel for immunoblot analysis. Statistical significance: * *p* < 0.05, ** *p* < 0.01, *** *p* < 0.001.

**Figure 6:**
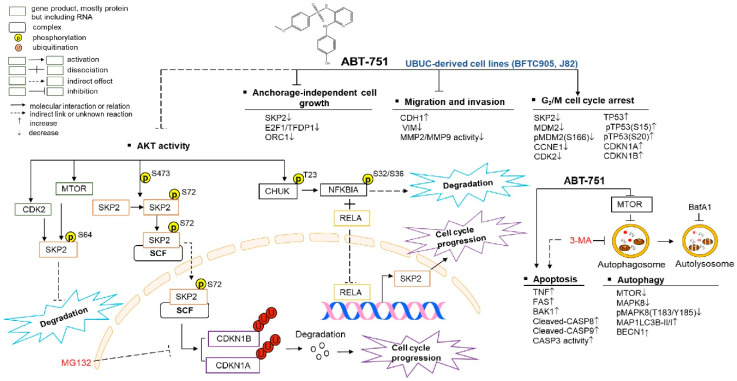
Summary of this study.

To evaluate whether exogenous *SKP2* expression restored the effects of ABT-751 on cell proliferation, we overexpressed the *SKP2* gene in BFTC905 and J82 cells without or with ABT-751 treatment. Overexpression of the *SKP2* gene remarkably upregulated SKP2-HaloTag fusion, stable forms of SKP2 (pSKP2(S72) and pSKP2(S64)), while it downregulated CDKN1A, CDKN1B protein levels (Figure 5A) meanwhile decreased cell proliferation (Figure 5B) in both cell lines. The treatment of ABT-751 in *SKP2* transfectants for 24 h notably downregulated pSKP2(S72), pSKP2(S64) whereas it upregulated CDKN1A and CDKN1B protein levels (Figure 5A) and downregulated SKP2-induced cell proliferation (*p* < 0.001, Figure 5B). To further determine whether ABT-751 treatment suppressed SKP2 protein level also through inhibition of AKT signaling, exogenous expression of the constitutive active *AKT*1 gene without or with ABT-751 treatments was performed in BFTC905 and J82 cells. Overexpression of the active *AKT*1 gene markedly upregulated exogenous AKT1, pAKT1(S473), pCHUK(T123), NFKBIA, pNFKBIA(S32/S36), RELA, pSKP2(S72), however it downregulated CDKN1A and CDKN1B protein levels. The treatment with ABT-751 for 24 h further downregulated AKT1-induced pAKT1(S473), pCHUK(T23), NFKBIA, pNFKBIA(S32/S36), RELA, pSKP2(S72) whilst upregulating AKT1-suppressed CDKN1A and CDKN1B protein levels (Figure 5C). Exogenous expression of the *AKT*1 gene induced cell proliferation (*p* < 0.001); nevertheless, ABT-751 treatment for another 24 h suppressed AKT1-induced cell proliferation (*p* < 0.001) (Figure 5D). Accordingly, ABT-751 suppressed SKP2 protein levels via the inhibition of phospho-AKT (post-translational) signaling. ABT-751 induced anticancer effects and its underlying molecular mechanisms are summarized in Figure 6.

## 3. Discussion

In this study, we provided considerable evidence to show that an anti-microtubule agent, ABT-751, induced profound anti-cancer effects, specifically the upstream cytostasis through the inhibition of *SKP2* mRNA and its corresponding protein levels. SKP2 is a well-recognized F-box protein; it is repeatedly overexpressed in several human carcinomas and impacts in tumorigenesis [24]. It has been shown that the increased *SKP2* mRNA levels contributed to the progression and predicted poor prognosis in human urothelial carcinoma [25]. Being a critical part of the SKP/Cullin/F-box-SKP2 (SCF^SKP2^) ubiquitin E3 ligase complex, SKP2 recruits specific substrate proteins for their ubiquitination and subsequent degradation by the 26S proteasome. Major substrates of SKP2 contain CDKN1A, CDKN1B, CDKN1C and E1A binding protein p300 (EP300) [26]. Belonging to the CIP/KIP family, CDKN1A, CDKN1B and CDKN1C bind and inhibit key regulators of the cell cycle cyclin–CDK complexes. On the other hand, SKP2 binds to the transcription cofactor EP300, which equips acetyl transferase activity and avoids TP53 acetylation, thereafter suppressing TP53-induced apoptosis [27]. Cell cycle dysregulation by downregulation or mutation in a series of CKIs including *CDKN*1*A* is a transition event where low-grade non-invasive papillary tumors progress to high-grade invasive UBUCs [28]. We herein identified that ABT-751 is an attractive *SKP2* inhibitor in UBUC-derived cells.

We additionally identified that ABT-751 inhibited *SKP2* transcription through the suppression of the AKT–CHUK–NFKBIA–NFKB signaling pathway. Earlier studies demonstrated that AKT activates phosphorylates CHUK (pCHUK(T23)) which further phosphorylates NFKBIA (pNFKBIA(S32/S36)), resulting in proteasomal degradation of the phospho-NFKBIA proteins and liberation of pNFKBIA-bound NFKBs such as the NFKB1–RELA complex. This complex is next translocated to the nucleus to transactivate target genes involving in cell growth including *SKP2* [29,30,31]. In the present study, ABT-751 downregulated pAKT1(S473) (active AKT1), AKT1 kinase activity and pCHUK(T23) (AKT1 substrate). Two phosphorylated-NFKBIA (S32/S36), a substrate of pCHUK(T23) and Y42 were phosphorylated by ABT-751-induced reactive oxygen species [18,19] and downregulated nuclear RELA. However, it maintained a robust interaction between NFKIBA and RELA, limiting RELA nuclear translocation and *SKP2* transcription. In the AKT–CHUK–NFKBIA–NFKB pathway, the protein expression pattern treated by ABT-751 was quite similar to that of treatment with a PI3K-AKT inhibitor, LY294002, suggesting that ABT-751 may be also a PI3K-AKT inhibitor.

In addition to downregulating *SKP2* at the mRNA level, ABT-751 may also regulate SKP2 by ATK1 and MTOR or CDK2 at posttranslational levels, because two stable forms of SKP2: pSKP2(S72) and pSKP2(S64) were meaningfully downregulated after ABT-751 treatment. Recent biochemical experiments disclosed that SKP2 can be phosphorylated by AKT1 and CDK2 or MTOR at residues S72 and S64 [32,33,34]. The phosphorylation of SKP2 on these residues avoids FZR1 (complexed with CDC20, a component of multi subunit RING-finger type E3 ligase targeting SKP2 for degradation [35]) binding to SKP2, thus mitigating SKP2 ubiquitination and degradation [23]. The treatment of MG132 alone downregulated SKP2 protein levels, similar to an earlier report, due to enhance ubiquitination [34]. Interestingly, treatment with MG132, further decreased ABT-751-suppressed cytosolic or nuclear SKP2 protein levels, suggesting that ABT-751-inhibited SKP2 is at least partially due to proteasome-mediated degradation. Therefore, except for FZR1 and CDC20, more specific E3 ligase(s) targeting SKP2 for degradation may exist. Moreover, ABT-751 treatments did not increase FZR1 or CDC20 levels. It downregulated/destabilized pSKP2(S72) and pSKP2(S64), potentially by the inhibition of several kinases. Due to ABT-751-induced DNA damage [18,19] which may inhibit CDK2 [36], AKT [37] and MTOR [38] activities, the probability of ABT-751-inhibited kinases and downstream phosphorylated SKP2 was elevated, suggesting that ABT-751 is a potential inhibitor of multiple kinases, consistent with former research regarding to other microtubule-binding agents [39].

ABT-751 also blockaded cell migration, invasion, MMP2/MMP9 activity along with upregulation of CDH1 and downregulation of VIM protein levels in vitro. A main effect of MMPs in tumor migration is to degrade extracellular matrix; therefore, original tumor cells metastasize. Particularly, MMP2/9 degrade type IV collagen, the key element of the basement membrane. Type IV collagen also serves the purpose of maintaining tissue organization and the structure of cells. An indispensable step for the metastatic progression of most cancers is degradation of the basement membrane [40]. Loss of *CDH*1 function is believed to confer tumorigenicity by increasing proliferation, invasion, and/or metastasis. Cell migration is promoted by VIM upregulation through assimilating environmental mechanical inputs, as well as restraining microtubules and actomyosin network dynamics. During mitosis, microtubules direct intracellular trafficking of proteins, organelles, vesicles and separate chromosomes. These organelles are essential for cell proliferation, shaping, cell migration, secretory processes and vascularization [41]. Hence, in addition to inhibiting cell proliferation, ABT-751 suppressed cell migration and invasion accompanied with the inactivation of MMP2/9 enzymes. These results are consistent with another study using one ABT-751-derived compound, N-[2(4-Methoxyphenyl)ethyl]-1,2-dihydro-pyrimidine [2,1-b] quinazolin-6-one (16a) [42].

Similar to other anti-microtubule agents [43], ABT-751 induced autophagy in BFTC-905 and J82 cells. To our surprise, among numerous molecules involving the initiation and process of autophagy [44,45], ABT-751-downregulated MTOR protein levels were sufficient to induce autophagy as evident by the increase in CytoID, MAPLC3II/I ratio and BECN1. We further identified that intrinsic and extrinsic apoptosis were rapidly induced after ABT-751 treatments in two distinct UBUC-derived cells, accompanied with alteration on the expression levels of specific markers. Undoubtedly, treatment with anti-microtubule agents such as ABT-751 induced DNA damage in hepatocellular carcinoma-derived cells [18,19], which is strong stress to cells. Thereafter, this treatment upregulated and stabilized TP53, consistent with an earlier report regarding autophagy is part of the protective function of TP53 [46]. On the other hand, ABT-751 treatment may act like other microtubule destabilizing agents through the inhibition of tubulin which activates pMAPK8(T183/Y185) but suppresses active/phosphorylated AKT1 and downstream mechanistic target of rapamycin complex 1 (mTORC1), including MTOR kinase [39] and induces autophagy. Insufficient apoptosis is the major hurdle for cancer treatment. Since autophagy occurred as early as 2 h after ABT-751 treatment, inhibition of early (autophagosome formation) rather than late (autolysosome) autophagy became a rational strategy to enhance the apoptotic effect of this drug.

Indeed, in advanced or metastatic NSCLC, a phase I/II study of pemetrexed (an inhibitor of thymidylate synthase dihydrofolate reductase and glycinamide ribonucleotide formyltransferase) with or without the combination of ABT-751 treatment was not able to improve the progression-free survival. However, difference in overall survival (*p* = 0.034, log-rank; median 3.3 vs. 8.1 months) favored ABT-751 in the squamous NSCLC subgroup. Therefore, patient stratification is critical to achieve success in clinical trials [14]. Our preclinical experiments showed promising anticancer efficacy of ABT-751 in distinct UBUC-derived cells. Nevertheless, patient stratification based on specific markers in subtype UBUC may improve clinical outcomes.

Overall, we identified that ABT-751 inhibited cell proliferation, cell migration and invasion accompanied with alternated expression levels of several cell cycle regulators including SKP2, CDK2, MTOR and two EMT-related proteins, CDH1 and VIM, in two distinct UBUC-derived cell lines, BFTC905 and J82. As early as 2 h after ABT-751 treatment, autophagy was induced. Therefore, the inhibition of autophagy took place at early rather than late stage augmented apoptosis. ABT-751 inhibited SKP2 protein level was ubiquitin–proteasome-irrelevant, however, it suppressed *SKP2* at both transcriptional and post-translational levels through the AKT–CHUK–NFKBIA–NFKB (RELA) axis and acted like a PI3K-AKT inhibitor. Therefore, we identified this microtubule agent, ABT-751, that induced cytostasis through the inhibition of the expression of SKP2 in UBUC-derived BFTC905 and J82 cells.

## 4. Materials and Methods

### 4.1. Cell Culture

The human UBUC-derived cell lines, BFTC905 [47] (Bioresource Collection and Research center, Hsinchu, Taiwan), and J82 (ATCC, Manassas, VA, USA), were, respectively, cultured in Roswell Park Memorial Institute (RPMI) 1640 (ThermoFisher Scientific, Waltham, MA, USA) and Dulbecco’s Modified Eagle’s Medium (DMEM, GE Healthcare HyClone^®^) media containing 15% and 10% (v/v) fetal bovine serum (FBS) (Biological Industries, Beit, HaEmek, Israel Israel), 50 IU/mL penicillin, 50 μg/mL streptomycin (HyClone, Logan, UT, USA), 1% sodium pyruvate, 1% nonessential amino acids and 1.5 g/L sodium bicarbonate. Both cell lines were maintained in a humidified incubator with 5% CO_2_ at 37 °C. The BFTC905 and J82 cell lines were characterized to embrace a wild type and multiple mutations of the *tumor protein p*53 (*TP*53) gene, respectively [48].

### 4.2. Chemicals

We obtained ABT-751 from ShangHai Biochempartner Co., Ltd. (Shanghai, China). A stock solution was prepared by dissolving ABT-751 in 50 mM dimethyl sulfoxide (DMSO). A concentrated solution was prepared by diluting with a final concentration of 1/1000 DMSO in cell culture media for each experiment. We purchased all chemicals from Sigma-Aldrich unless stated otherwise.

### 4.3. Cell Viability Assay

For cell viability analysis, the MTT assay was used to identify the half maximal inhibitory concentration (IC_50_) of ABT-751 in BFTC905 and J82 cells. Cells were seeded in 96-well plates and treated with various concentrations of ABT-751 at 37 °C in a humidified atmosphere with 5% CO_2_ at the indicated time points. After treatments, cells were incubated in culture medium containing MTT (final concentration 0.5 mg/mL) for 4 h at 37 °C. Subsequently, the supernatant was removed and 100 µL of DMSO were added to each well. The absorbance was measured at 570 nm.

### 4.4. Flow Cytometric Assays

Cell cycle analysis: cells (BFTC905, 1 × 10^6^ and J82, 5 × 10^5^) were treated with the indicated concentration of ABT-751 (BFTC905, 0.6 µM and J82, 0.7 µM) or DMSO (control) for 24 h. Cells were collected, washed with ice-cold Phosphate-Buffered Saline (PBS), fixed with 70% ethanol and stored overnight at −20 °C. Before analysis, cells were washed with ice-cold PBS twice and resuspended in a 1 mL mix of 200 µg/mL RNAse and 50 µg/mL propidium iodide (PI) in PBS and incubated for 30 min at 37 °C. Samples were analyzed by flow cytometry (NovoCyte™ 2000, ACEA, Brussels, Belgium) and NovoExpression™ software (Version 1.2.1, ACEA).

CytoID^®^ fold change (autophagy): The Cyto-ID^®^ Autophagy Detection Kit (ENZ-51031, Enzo Life Sciences, Farmingdale, NY, USA) was used to determine autophagic vacuoles and monitor autophagic flux. Cells were treated with the indicated concentration of ABT-751 for 24 h. Starvation (media containing 2% fetal bovine serum) served as a positive control. After treatments, cells were washed with 1 × Assay Buffer, and the diluted Cyto-ID^®^ green stain solution was added to each sample for 30 min at room temperature in the dark. Then cells were washed with 1 × Assay Buffer and immediately analyzed by flow cytometry.

Apoptosis: cells were treated with the indicated concentration of ABT-751 or DMSO (control) for 24 h, then cells were centrifuged and resuspended in eBioscience™ Annexin V Binding Buffer (Annexin V-FITC Apoptosis Detection Kit, #BMS500F1, ThermoFisher Scientific). Annexin V with fluorescein (FITC)-labeled nucleotides as well as PI staining was performed following the manufacturer’s instructions. We utilized flow cytometry for measuring apoptotic cells.

### 4.5. Soft Agar Assays

A CytoSelect™ 96-well in vitro tumor sensitivity assay (soft agar colony formation, CBA-150, CELL BIOLABS, Inc. San Diego, CA, USA) was used to analyze whether treatments with ABT-751 affected colony formation and anchorage-independent cell growth in BFTC905 and J82 cells according to our previous study [49]. The incubation periods were 7 days after treatment with DMSO (control) or ABT-751 (BFTC905, 0.6 μM and J82, 0.7 μM). The MTT assay was used to quantitate the anchorage-independent cell growth.

### 4.6. Immunoblot Assay, Antibodies and Nuclear/Cytosol Fractionation

Immunoblot analyses were performed similar to our previous study [18]. Briefly, cell lysates were prepared with Radioimmunoprecipitation assay (RIPA) buffer (#20-188, Merck Millipore, Burlington, MA, USA). Lysates containing equal amount of protein were separated by 8, 10, 12 or 15% SDS-PAGE as appropriate and electroblotted onto the FluoroTrans^®^ Polyvinylidene Fluoride (PVDF) Transfer Membrane (Pall, New York, NY, USA). The filters were individually probed with the primary anti-human antibodies (1:1000 for all antibodies). Detailed information of antibodies is provided in the Supplementary Materials and Methods. To monitor the nuclear protein levels after treatment with a proteasome inhibitor MG132 for 6 h and/or ABT-751 for another 18 h and ABT-751 for 24 h, respectively, nuclear/cytosol fractionation along with immunoblot analysis were performed. BFTC905 cells (2.5 × 10^6^) were seeded in 10-cm culture dishes and treated with DMSO (control), MG132 or/and ABT-751, respectively. One Nuclear/cytosol Fractionation Kit (K266-25, BioVision Inc. Milpitas, CA, USA) was applied to isolate nucleus and cytosol using trypsinized cells according to the manufacturer’s protocol.

### 4.7. Transwell Migration, Transwell Invasion and Matrix Metallopeptidase 2/9 Activity Assays

We analyzed cell migration and invasion using the QCM ECMatrix Cell Invasion Kit (ECM554, Merck Millipore). A transwell invasion assay was performed in chambers which were freshly coated with Matrigel (BD Biosciences, San Jose, CA, USA) based on the manufacturer’s protocol. Cells (5 × 10^4^) treated with ABT-751 and DMSO (control) were starved in media containing 2% FBS at 37 °C overnight. Next, cells were plated in the top chamber on the Matrigel-coated membrane in serum free media. Media containing 15% FBS were added into the lower chambers, which were used as a chemoattractant, and the cells were incubated for another 24 h. Non-invasive cells were removed from the upper surface of the membrane with a cotton swab. The penetrated cells were detached with Cell Detachment Solution and lysed with Lysis Buffer/Dye Solution. The lysed mixtures were transferred to a 96-well plate for fluorescence measurement at wavelengths excitation/emission: 480/520 nm using a GloMax^®^-Multi+ Detection System (Promega, Madison, WI, USA). Transwell migration was analyzed by the same procedure, except cells were plated in the top chamber on the non-coated membrane.

For matrix metallopeptidase (MMP) activity assay, we used the MMP2/MMP9 Solution Assay Kit (#E-118SA, Biomedical Research Service Center, University at Buffalo, State University of New York) based on MMP2/9-mediated hydrolysis of blue gelatin in solution. Cells (1.5 × 10^6^) were seeded overnight and treated with DMSO (control) or ABT-751 (BFTC905, 0.6 μM and J82, 0.7 μM) for 24 h. 5 × MMP Buffer, Blue Gelatin and media collected from control (DMSO) and test (ABT-751 treatment) were mixed sequentially and pipetted thoroughly. The solutions were incubated at 37 °C overnight in an Intelli-Mixer Rotomixer (RM-2). We used Ice-cold acetone to stop the reaction, subjected it to vortex and mixed completely; the material was kept on ice for protein precipitation for 10 min. Samples were next centrifuged. Exactly 100 μL of supernatants were moved to a 96-well plate and read with the wavelength of OD595 nm using a GloMax^®^-Multi + Reader (Promega, Fitchburg, WI, USA).

### 4.8. Quantitative Reverse Transcription-Polymerase Chain Reaction

Quantitative reverse transcription-polymerase chain reaction (RT-PCR) was applied to quantify the mRNA expression levels of several genes using predesigned TaqMan^®^ reagents from ThermoFisher (*SKP2*: Hs01021864_m1 (59 bp); *TP*53: Hs01034249_m1 (108 bp); *CDKN*1*A*: Hs00355782_m1 (66 bp); *CDKN*1*B*: Hs01597588_m1 (151 bp); *RB*1: Hs01078066_m1 (72 bp); *E*2*F*1: Hs00153451_m1 (84 bp); *TFDP*1: Hs00955488_g1 (102 bp); *glyceraldehyde-*3*-phosphate dehydrogenase* (*GAPDH*): Hs02758991_g1 (93 bp); *CD*44 *molecule, Indian blood group* (*CD*44): Hs01075861_m1 (70 bp); *cadherin* 1 (*CDH*1): Hs01023895_m1 (80 bp); *vimentin* (*VIM*): Hs00185584_m1 (73 bp); *MTOR*: Hs00234508_m1 (103 bp)) along with LightCycler^®^ 96 System (Roche, Basel, Switzerland) and ΔΔC_T_ calculation. Briefly, total RNAs were extracted with TRIzol^®^ reagent (ThermoFisher) from cells and reverse-transcribed using the High Capacity cDNA Reverse Transcription Kit (ThermoFisher). The relative expression folds of target transcripts were given by 2^−ΔΔCT^, where ΔΔC_T_ = ΔC_T(treatment)_ − ΔC_T(control)_. ΔC_T(treatment)_ = C_T(target)_ − C_T(GAPDH)_ and ΔC_T(control)_ = C_T(target)_ − C_T(GAPDH)_.

### 4.9. Colorimetric Assay of the Caspase 3 Activity

In addition to immunoblot analysis on cleaved-caspase 8 (CASP8) and -CASP9, caspase 3 (CASP3) activity was further evaluated with the Caspase 3/CPP32 Colorimetric Assay Kit (#K106-25, BioVision). This assay is based on spectrophotometric detection of the chromophore *p*-nitroaniline (*p*NA) after cleavage from the labeled substrate aspartic acid-glutamic acid-valine-aspartic acid (DEVD)-pNA. Briefly, cells (1.5 × 10^6^) were treated with DMSO (control) or ABT-751 for 24 h, resuspended in 50 μL of chilled Cell Lysis Buffer and incubated on ice for 10 min. Samples were next centrifuged with 10,000× *g* for 10 min and the supernatants (cytosolic extract) were transferred to fresh tubes on ice and protein concentrations were next measured using the Bradford assay. For each assay, protein (150 μg/each) was diluted with 50 μL Cell Lysis Buffer, 50 μL of 2× Reaction Buffer (containing 10 mM dithiothreitol (DTT), 5 μL of the 4 mM DEVD-pNA substrate (200 μM final concentration) were added and incubated at 37 °C for 2 h. The *p*NA light emission was quantified using a GloMax^®^ − Multi + Reader (Promega) with the excitation/emission: 405 nm/495 nm.

### 4.10. AKT Activity Assay and Co-Immunoprecipitation

The AKT Activity Assay Kit (KinaseSTAR, #K435, Biovision) based on co-immunoprecipitation technology was used according to the manufacture’s instructions. Similarly, to examine whether RELA interacts with NFKB inhibitor alpha (NFKBIA), co-immunoprecipitation was performed using our previous procedure [49] with some modifications. Cells were trypsinized after DMSO (control) or ABT-751 treatment for 24 h. Normal rabbit IgG (1:50, NI01, Millipore, Burlington, MA, USA) and anti-RELA antibody (1:50) pre-conjugated to protein G agarose beads (#K286-25, BioVision) were subjected to SDS-PAGE and immunoblotted with anti-RELA and anti-NFKBIA antibodies as described in immunoblot analysis.

### 4.11. Plasmids and Transient Transfection

Two plasmids, pCMV10-3xFlag-SKP2(WT) (#81115) and pHRIG-AKT1 (#53583, constitutive expression of the active *AKT*1 gene), were obtained from addgene (Watertown, MA, USA). The *SKP2* gene was subcloned into pHaloTag using the In-Fusion^®^ HD Cloning Kit (#121416, Takara, Mountain View, CA, USA) based on the manufacturer’s instructions. The primers used for subcloning were 5′-TCACTATAGGGCTAGCATGCACAGGAAGCACCTCCAG-3′ and 5′-CAGTGGTTGGCTCGAGTAGACAACTGGGCTTTTGCAGTGT-3′. Cells were seeded in 6-cm dishes overnight and transfected with 2.5 μg of pHaloTag (control), pSKP2-HaloTag or pHRIG-AKT1 plasmid by mixing with 3 μL PolyJet™ reagent for 16 h, replaced with normal medium and incubated for another 8 h before analysis.

### 4.12. Cell Proliferation Assay

To examine whether ABT-751 suppressed cell proliferation, a Cell Proliferation Assay Kit (Fluorometric, #K307-1000, BioVision) was used. It is based on a nuclear dye that specifically binds to nucleic acid in the cell and generates green fluorescence. The generated fluorescent intensity is directly proportional to the cell number, which can be quantified by measuring fluorescence (excitation/emission: 480 nm/538 nm). Shortly, cells (5 × 10^3^) were seeded in a 96-well plate with 100 μL of culture medium overnight, transfection of pSKP2-HaloTag or pHRIG-AKT1 was performed for 18 h, treated with DMSO (control) or ABT-751 for another 24 h. Precisely 25 μL of 5 × Nuclear Dye/Cell Lysis Buffer was added into each well and gently shaken with a digital rotator at room temperature protected from light for 15 min and subjected to be analysis.

### 4.13. Statistical Analysis

All data were confirmed at least three times independently. Normal distributed data were expressed as the means ± SD. The data values lying outside two SD of the means (mean ± 2SD, ~95%) were considered as outliers. Statistical analysis was performed using Statistical Package for the Social Sciences (SPSS) v.18.0 (IBM, Armonk, NY, USA). Differences between the two groups were analyzed by the Student’s *t*-test. Differences among ≥ 3 groups were analyzed by one-way or two-way analysis of variance as appropriate, followed by the Scheffe multiple comparison test. A *p-*value < 0.05 is considered to be of statistical significance.

## Data Availability

The data are shown in the main manuscript, supplementary documentation and available to readers.

## Supplementary Materials

The Supplementary Materials are available online at https://www.mdpi.com/1422-0067/22/2/945/s1.
