# Supplementary material for: ABT-751 Induces Multiple Anticancer Effects in Urinary Bladder Urothelial Carcinoma-Derived Cells: Highlighting the Induction of Cytostasis through the Inhibition of SKP2 at Both Transcriptional and Post-Translational Levels
Source: Int J Mol Sci. 2021 Jan 19;22(2):945. doi: 10.3390/ijms22020945 (PMC7835924; doi:10.3390/ijms22020945)
Supplement: Supplementary file 1 [file ijms-22-00945-s001.pdf]

Supplementary Documentation

Supplementary Materials and Methods

Antibodies including anti-S-phase kinase associated protein 2 (SKP2, #32-3300, ThermoFisher Scientific), -MDM2 proto-oncogene (MDM2, sc-965, Santa-Cruz Biotechnology, Dallas, TX, USA), -phospho-MDM2(serine 166) [pMDM2(S166)] (#3521, Cell Signaling Technology, Denver, MA, USA), tumor protein p53 (TP53) (#9282, Cell Signaling), -pTP53(S15) (#9286, Cell Signaling), -pTP53(S20) (#9287, Cell Signaling), cyclin dependent kinase inhibitor 1A (CDKN1A) (#2947, Cell Signaling), cyclin dependent kinase inhibitor 1B (CDKN1B) (#3686, Cell Signaling), cyclin dependent kinase inhibitor 1C (CDKN1C) (#sc-1040, Santa Cruz), cyclin D1 (CCND1, #2926, Cell Signaling), -cyclin E1 (CCNE1) (sc-198, Santa Cruz), -cyclin-dependent kinase 2 (CDK2, sc-163, Santa Cruz), -cyclin A2 (CCNA2) (GTX103042, GeneTex, Irvine, CA, USA), -RB transcriptional corepressor 1 (RB1, sc-102, Santa Cruz), -E2F transcription factor 1 (E2F1, sc-251, Santa Cruz), -transcription factor Dp-1 (TFDP1) (sc-53642, Santa Cruz), - cyclin dependent kinase 9 (CDK9) (GTX107758, GeneTex), -chromatin licensing and DNA replication factor 1 (CDT1) (GTX109663, GeneTex), -origin recognition complex subunit 1 (ORC1) (Ab138102, Abcam, Cambridge, UK), pan-actin (MAB1501, Merck Millipore), -cell division cycle 20 (CDC20) (GTX111137, GeneTex), -fizzy and cell division cycle 20 related 1 (FZR1) (GTX111200, GeneTex), -AKT serine/threonine kinase (pan-AKT) (#4691, Cell Signaling), -pAKT1(S473) (#4060, Cell Signaling), phospho-component of inhibitor of nuclear factor kappa B kinase complex [pCHUK(T23), ab38515, abcam)], -NFKB inhibitor alpha (NFKBIA) (GTX110521, GeneTex), -pNFKBIA(S32/S36) (#9246, Cell Signaling), -RELA proto-oncogene, NFKB subunit (RELA, also known as p65) (#6956, Cell Signaling), -pNFKBIA(Tyr42) (GTX50681, GeneTex), -phospho-glycogen synthase kinase-3 beta [pGSK3B(S9)] (#5558, Cell Signaling), -Myc proto-oncogene, bHLH transcription factor (c-Myc) (GTX103436, GeneTex), -forkhead box O3 (FOXO3) (ab12162, Abcam), -pFOXO3(S253) (#9466, Cell Signaling), -cAMP responsive element-binding protein 1 (CREB-1) (sc-186, Santa-Cruz), -pCREB-1(S133) (#06-519, Merck Millipore), -glyceraldehyde-3-phosphate dehydrogenase (GAPDH) (ab128915, Abcam), -cadherin 1 (CDH1) (#610181, BD Biosciences, San Jose, CA, USA), -CD44 molecule (Indian blood group) (CD44) (15675-1-AP, Proteintech, Rosemont, IL, USA), -vimentin (VIM) (#3932, Cell Signaling), -tumor necrosis factor (TNF) (GTX110520, GeneTex), -TNF receptor superfamily member 1A (TNFRSF1A) (GTX102710, GeneTex), -Fas cell surface death receptor (FAS) (sc-715, Santa Cruz), -Fas associated via death domain (FADD) (GTX116064, GeneTex), -BCL2 associated X, apoptosis regulator (BAX) (sc-493, Santa Cruz), -BCL2 antagonist/killer 1 (BAK1) (#3814, Cell Signaling), -BCL2 apoptosis regulator (BCL2) (#2872, Cell Signaling), -caspase 8 (CASP8) (#9496, Cell Signaling), -caspase 9 (CASP9) (#9508, Cell Signaling), -DNA damage regulated autophagy modulator 2 (DRAM2) (#MABC64, Merck Millipore), -mechanistic target of rapamycin kinase (MTOR) (GTX101557, GeneTex), -mitogen-activated protein kinase 8 (MAPK8, also known as JNK, Ab110724, Abcam), -pMAPK8(T183/Y185) (#9251, Cell Signaling), -sequestosome 1 (SQSTM1) (sc-28359, Santa Cruz), -autophagy related 5 (ATG5) (GTX102361, GeneTex), -autophagy related 12 (ATG12) (GTX124181, GeneTex) and -beclin 1 (BECN1) (GTX113039, GeneTex) antibodies were used. Protein bands were detected by FemtoGlow™ Stable Western PLUS (#FWPD03-500, Michigan Diagnostics,

Royal Oak, MI, USA) with horseradish-peroxidase-conjugated secondary antibody [anti-mouse (#11503062) or anti-rabbit (#11503045), Jackson ImmunoResearch] and visualized on a ChemiDoc™ XRS+Imaging System (#12003153, Bio-Rad, Hercules, CA, USA).

Supplementary Figures

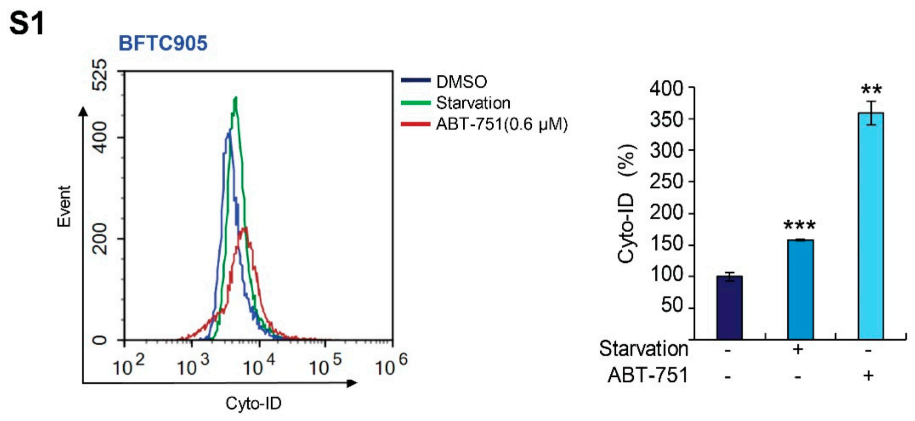

Figure S1. Cells were treated with the indicated concentration of ABT-751 (0.6 μM) for 24 h in BFTC905 cells, then autophagic flux was determined by Cyto-ID® Autophagy Detection Kit, starvation (containing 2% FBS in culture medium) served as a positive control.

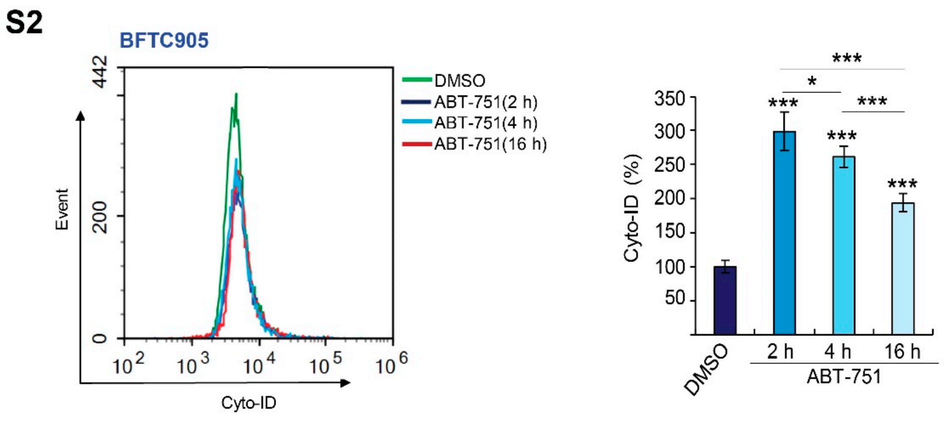

Figure S2. ABT-751 stimulated autophagy in time-dependent manners. Cells were treated with the indicated concentration of ABT-751 (0.6 μM) in time-dependent manners, then autophagic flux was determined by Cyto-ID® Autophagy Detection Kit.

S3

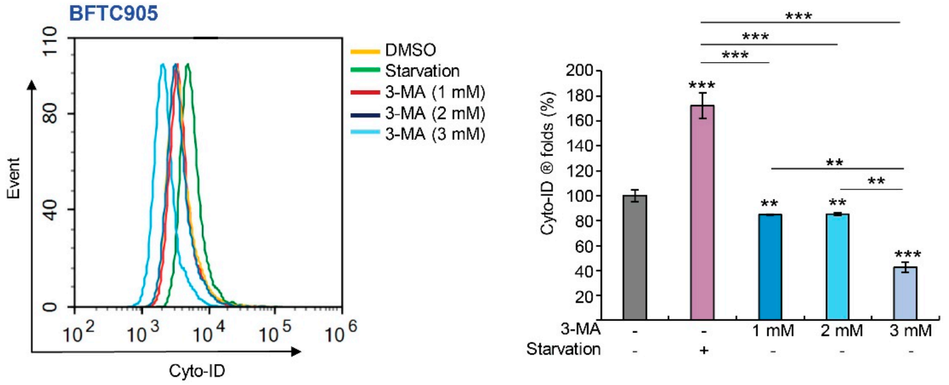

Figure S3. 3-methyladenine (3-MA, an autophagosome inhibitor) inhibited endogenous autophagy in a dose-dependent manner.

S4

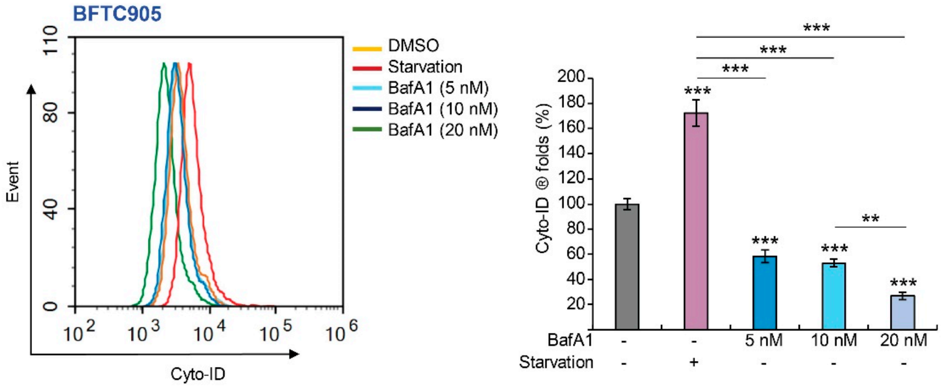

Figure S4. Bafilomycin A1 (BafA1, an autolysosome inhibitor) inhibited endogenous autophagy in a dose-dependent manner.

S5

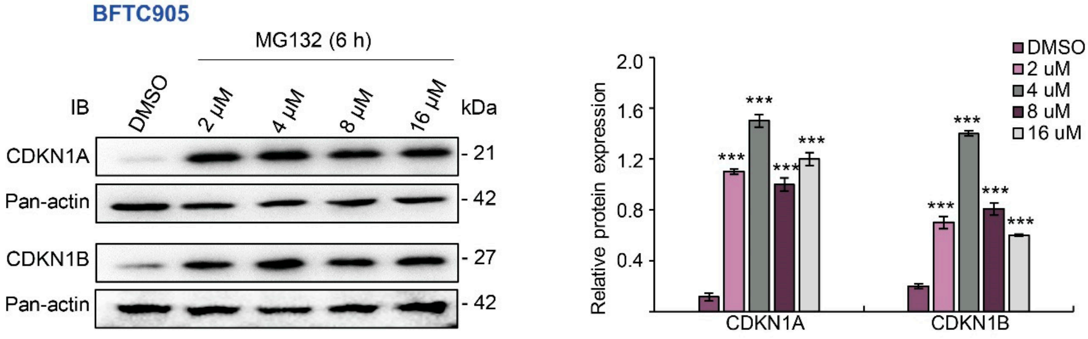

Figure S5. Cells were treated with different concentration of the MG132 (a proteasome inhibitor) for 6 h, then the level of endogenous CDKN1A and CDKN1B proteins were determined by immunoblots.
